# Extraintestinal Manifestations in Inflammatory Bowel Disease: From Pathophysiology to Treatment

**DOI:** 10.3390/biomedicines12081839

**Published:** 2024-08-13

**Authors:** Ilaria Faggiani, Jacopo Fanizza, Ferdinando D’Amico, Mariangela Allocca, Alessandra Zilli, Tommaso Lorenzo Parigi, Alberto Barchi, Silvio Danese, Federica Furfaro

**Affiliations:** 1 Department of Gastroenterology and Endoscopy, IRCCS San Raffaele Hospital, 20132 Milan, Italy; faggiani.ilaria@hsr.it (I.F.); fanizza.jacopo@hsr.it (J.F.); damico.ferdinando@hsr.it (F.D.); allocca.mariangela@hsr.it (M.A.); zilli.alessandra@hsr.it (A.Z.); parigi.tommaso@hsr.it (T.L.P.); sdanese@hotmail.com (S.D.); furfaro.federica@hsr.it (F.F.); 2 Gastroenterology and Endoscopy, Vita-Salute San Raffaele University, 20132 Milan, Italy

**Keywords:** extraintestinal manifestation, Crohn’s disease, ulcerative colitis, inflammatory bowel diseases

## Abstract

The inflammatory bowel diseases (IBDs) are systemic conditions that affect not only the gastrointestinal tract but also other parts of the body. The presence of extraintestinal manifestations can significantly impact the quality of life in IBD patients. Peripheral arthritis, episcleritis, and erythema nodosum are frequently associated with active intestinal inflammation and often improve with standard treatment targeting intestinal inflammation. In contrast, anterior uveitis, ankylosing spondylitis, and primary sclerosing cholangitis typically occur independently of disease flares. The incidence of these conditions in individuals with IBD can reach up to 50% of patients over the course of their lifetime. In addition, some advanced therapies utilized for the treatment of IBD potentially result in side effects that may resemble extraintestinal manifestations. This review provides a thorough analysis of the pathophysiology and treatment of extraintestinal manifestations associated with Crohn’s disease and ulcerative colitis.

## 1. Introduction

Inflammatory bowel diseases (IBDs) are chronic conditions including ulcerative colitis (UC) and Crohn’s disease (CD). IBDs are multifaceted conditions that impact patients’ lives. They require a comprehensive approach, considering not only their effects on the gastrointestinal system but also their potential extraintestinal manifestations (EIMs) [1].

EIMs can occur in up to 50% of patients with IBD, and they can be a significant cause of morbidity and, in some cases, mortality. Various organs can be affected, including the musculoskeletal, cutaneous, hepatic, ocular, and vascular systems [2]. The pathophysiology of EIMs is not yet fully understood. Possible driving forces include the extension of the immune-mediated response from the inflamed gut to other organs, changes in leukocyte trafficking, alterations to the intestinal microbiota, and underlying genetic predisposition [3].

Currently, there are numerous advanced therapies available that can effectively manage both intestinal diseases and EIMs. However, these drug side effects can sometimes be mistaken for EIMs, and the differential diagnosis is not always easy [4]. For this reason, UC and CD should be considered complex diseases that present a challenge for gastroenterologists to face within a multidisciplinary team.

In this review, we provide an overview of EIMs in IBD, from pathophysiology to treatment.

## 2. Materials and Methods

We conducted a comprehensive search of the Pubmed, Embase, and Scopus databases up until 30 May 2024, with the aim of identifying studies regarding extraintestinal manifestation in IBD. To achieve this, we employed specific search terms: ‘extraintestinal manifestations’, ‘musculoskeletal manifestations’, ‘cutaneous manifestations’, ‘ocular manifestations’, ‘hepatic manifestations’, in conjunction with ‘Crohn’s disease’, ‘ulcerative colitis’, and ‘inflammatory bowel disease’, ‘IBD’, ‘CD’, and ‘UC’. We limited our search to articles published in the English language.

Our screening process involved two independent reviewers (IF and FJ) who initially assessed titles and abstracts to identify potentially relevant studies. Subsequently, we examined the full texts of these selected articles to determine their eligibility for inclusion. Additionally, we manually scrutinized the reference lists of these articles to ensure that no relevant studies were overlooked during the electronic search. The final inclusion of abstracts and articles was based on their relevance to our research objectives.

## 3. Results

### 3.1. Pathophysiology of EIMs

Despite significant efforts, the pathogenesis of EIMs is not yet fully understood, given the complexity and multifactorial nature of these diseases. EIMs are characterized by either an antigen-specific immune reaction originating in the intestine and affecting a site outside the intestine, or an inflammatory process independent of intestinal involvement. These processes may be triggered or sustained by genetic or environmental factors in the host, or by the presence of IBD [3].

#### 3.1.1. EIMs as Extension of Immune Responses from the Intestine

A potential factor contributing to EIMs is the ectopical expression of chemokine and adhesion molecules in organs and tissues outside the gut. T cells are attracted to the gut due to the expression of α4β7 integrin and the CCR9 receptor, which bind to MAdCAM-1 and CCL25, respectively, expressed in the gut endothelium [5]. The altered lymphocytes homing in IBD can facilitate the upregulation of MAdCAM-1 and CCL25 expression in the liver and the migration of gut tropic T cells to sites outside of the intestine, contributing to the development of inflammation [6,7]. However, this can only partly explain EIMs, because this phenomenon does not occur in other organs, except for the liver [1].

**Figure 1:**
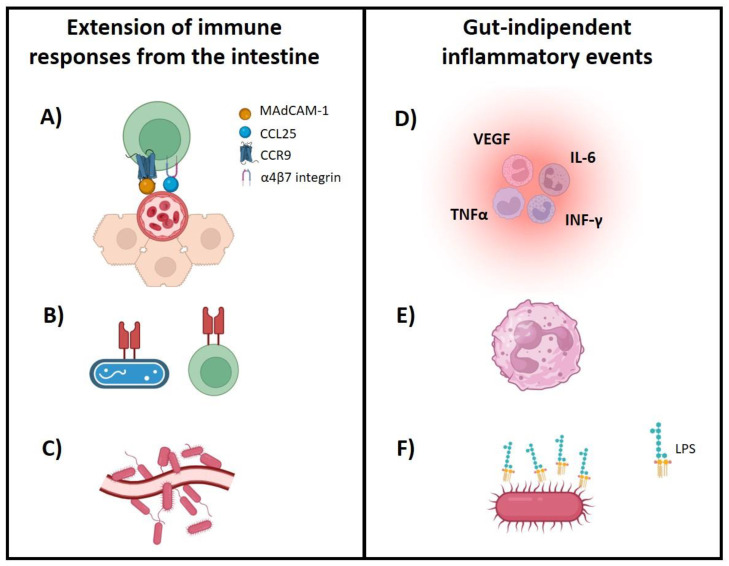
Potential pathophysiology of EIMs. Extension of immune responses from the intestine: (**A**) ectopic expression of adhesion molecules and chemokines, such as the abnormal expression of MAdCAM-1 and CCL25 in the vascular endothelium of the portal tract; (**B**) microbial antigen cross-reactivity, such as molecular mimicry, occurs between enteric bacteria and self-antigens presented by host’s major histocompatibility complex molecules; (**C**) microbial antigen translocation [8]. EIMs as independent inflammatory events: (**D**) shift in inflammatory tone influenced by genetic, environmental, or microbial factors, or by a systemic increase in key inflammatory mediators; (**E**) systemic changes in innate immune function, for example, neutrophil priming; (**F**) gut microbiota-induced distant inflammation driven by microbial products such as lipopolysaccharide, through changes in gut permeability and microbiota-derived metabolites [8]. MAdCAM-1: mucosal vascular addressin cell adhesion molecule 1; CCL25: chemokine (C-C motif) ligand 25; CCR9: C-C motif chemokine receptor 9; VEGF: vascular endothelial growth factor; IL-6: interleukin-6; TNFα: tumor necrosis factor α; INFγ: interferon γ; LPS: lipopolysaccharide.

Another theory proposes that EIMs may result from immune responses targeting antigens in the intestine but occurring outside of the intestinal tract due to cross-reactivity [8]. It has been demonstrated that enteric bacteria and host major histocompatibility complex molecules share peptide sequences [5]. The presence of a distinct microbiota in patients with primary sclerosing cholangitis (PSC) could confirm this hypothesis [9] (Figure 1).

#### 3.1.2. EIMs as Independent Inflammatory Events

The role of the microbiota in EIM is not yet fully defined. The potential role of molecular similarity between gut microbiota antigens and non-microbial epitopes on cells in EIM-affected organs in driving T-cell clone and immune cross-reactivity has not been definitively demonstrated [8]. Individuals with IBD and psoriatic arthritis (PsA) exhibit a relative decline in the prevalence of the *Coprococcus* and *Ruminococcus* species. These species play a crucial role in preserving the intestinal balance by generating short-chain fatty acids (SCFAs). SCFAs may have metabolic and immunomodulatory effects. Notably, in IBD patients with PsA, SCFAs are typically depleted [10]. Conversely, patients with IBD with spondyloarthritis (SpA) display elevated levels of the genus *Dialister*, a correlation that is positively linked to the activity of joint disease [11]. Finally, both IBD-associated arthropathy and rheumatoid arthritis (RA) patients have elevated levels of *Clostridiaceae*, suggesting a potential common microbial link in inflammatory arthritis [12].

Moreover, the alteration of the microbiota in patients with IBD has been observed to result in increased expression of lipopolysaccharide, thereby activating a number of proinflammatory molecules (interleukine-6, interferon γ and vascular endothelial growth factor) that could contribute to the development of EIMs [8] (Figure 1).

#### 3.1.3. Genetic Basis of EIMs

There is a significant overlap in genetic risk loci that are shared between IBD and EIMs [13,14,15,16] (Table 1). Interestingly, the identification of associated genetic mutations and polymorphisms of key immune pathways could allow for a molecular-based classification of EIMs, which would be more precise than an organ-based classification. This would also facilitate a more comprehensive understanding of the therapeutic targets, thereby enabling the administration of more effective therapy [17].

### 3.2. Musculoskeletal EIMs

Arthritis is the most frequent EIM of IBD, affecting axial joints, peripheral joints, or both [18]. SpA affects both sexes equally, with a higher prevalence in CD cases involving the colon than in UC, and it can occur before, simultaneously with, or after the onset of IBD [19]. Axial spondyloarthritis (axSpA) is typically characterized by symptoms such as back pain and morning stiffness of the spine. In contrast, peripheral spondyloarthritis (pSpA) is characterized by symptoms primarily affecting the upper and lower limbs [20]. Concerning prevalence, pSpA is the most common manifestation, occurring in 13% (95% CI: 12–15%) of cases, followed by sacroiliitis, at 10% (95% CI: 8–12%), and ankylosing spondylitis (AS), at 3% (95% CI: 2–4%) [18]. SpA is typically designated as ‘seronegative spondyloarthropathy’ due to the absence of rheumatoid factor (RF) positivity [21]. However, it is worth noting that SpA patients have a higher prevalence of positive RF test results compared to the general population (15% vs. 5%, respectively). Similarly, tests for anti-citrullinated peptide antibodies (ACPA) are typically negative, but they are positive more frequently than in the general population (8% to 12%, compared to 5%) [2,18,21].

AS commonly presents as nocturnal low back pain with morning stiffness, which is usually alleviated by physical activity, and it is often linked to uveitis. Conversely, sacroiliitis is asymptomatic in most patients [21]. The majority of patients with IBD affected by axSpA are positive for HLA-B27 (25–78%), and axial involvement can occur independently of gut pathology. It is more prevalent in CD compared to UC, with frequencies reaching up to 25% [22]. Both AS and sacroiliitis can be identified through magnetic resonance imaging (MRI) using the short-tau inversion recovery (STIR) technique [23].

pSpA can be categorized into two different types. Type 1 arthritis usually appears as acute, asymmetrical joint inflammation, affecting fewer than five joints, often involving the large knee joint [24]. It is associated with IBD activity and tends to resolve spontaneously within 10 weeks and shows a robust correlation with EIMs of IBD, such as erythema nodosum (EN) and uveitis [24]. Type 2 arthropathy is a symmetrical arthritis that affects five or more joints, often including the small metacarpophalangeal joint, and it is not associated with IBD activity and can persist for years [24].

Enthesitis, dactylitis, and tenosynovitis also occur in IBD patients as musculoskeletal EIMs [25]. Enthesitis can present as Achilles tendinitis, plantar fasciitis, or chest wall pain and is observed in 7% to 50% of adult IBD patients. Early detection can be aided by ultrasonography or MRI examination of the affected area [25].

### 3.3. Skin EIMs

Cutaneous manifestations are present in approximately 15–20% of patients with IBD [26]. Skin EIMs can be classified into four categories based on their pathophysiological mechanisms: skin manifestations that are reactive and have similar pathophysiological mechanisms to IBD (pyoderma gangrenosum (PG), EN, Sweet’s syndrome, aphthous ulcer); cutaneous manifestation that share the same histological features as IBD (metastatic CD); skin diseases related to IBD (psoriasis, hidradenitis suppurativa, and atopic dermatitis); skin lesions induced by IBD treatment [2].

EN, a septal panniculitis, is the most reported cutaneous manifestation in IBD (15%) and usually occurs during flare-ups of IBD. It is more common in CD (4–15%) than in UC (2.8–10%) [1]. Lesions are symmetric, raised, red, and nonulcerated subcutaneous nodules of 1–5 cm in size [27]. Typically, EN is self-limiting, with skin lesions usually resolving within 2–8 weeks without sequelae [27].

PG occurs in approximately 0.5–2.6% of patients with IBD and is the second most common EIM after EN [28]. It occurs more frequently in individuals with UC, particularly in females and the elderly [29], and may run parallel to IBD activity or follow an independent course [1]. Almost 15% of PG cases pre-date the onset of IBD [30]. It typically manifests as one or more tender, inflammatory papules or pustules that rupture quickly, and the ulcers can become necrotic and painful. The lesion size ranges from 2 to 20 cm and is characterized by violaceous undermined borders and peripheral erythema [26]. The most commonly affected areas are the extensor surfaces of the legs (70–80%) and areas adjacent to postsurgical stomas (18%) [28]. Diagnosis is typically based on clinical presentation, while skin biopsy, although it is not specific to this diagnosis, can be useful in ruling out other conditions (e.g., malignancies, skin infection) [31].

Sweet’s syndrome is a rare neutrophilic dermatosis that is associated with malignancies, infections, and, to a lesser extent, IBD [32]. It can manifest equally in UC and CD with erythematous papules and plaques involving the extremities, fever, and an elevation of systemic inflammatory markers [33]. The histologic feature that allows for the diagnosis is neutrophilic infiltration of the dermis without vasculitis. The management of the underlying IBD is resolutive, and systemic steroids are highly effective [2,33].

Aphthous stomatitis is characterized by multiple round or oval painful ulcers with a yellow pseudomembranous base and erythematous borders, typically found on the buccal or labial mucosa [27]. Diagnosis is clinical, although for persistent, recurrent, or refractory lesions, or in patients without a definitive IBD diagnosis, a biopsy of the ulcer borders and culture may be useful [34]. Aphthous stomatitis occurs in approximately 10% of patients with CD and UC, and in most cases, it is associated with exacerbations of IBD and the HLA-B27, so that controlling intestinal inflammation is decisive in most cases [35,36].

Metastatic CD is a rare skin EIM. Lesions usually affect the peri-genital area and they share the same characteristic of CD, such as non-caseating granuloma [26]. For mild forms, cutaneous treatments such as intralesional steroid injections and prolonged systemic metronidazole are recommended [2]. Tumor necrosis factor α (TNFα) antagonists and azathioprine (AZA) are second-line therapy [37].

### 3.4. Ocular EIMs

Ocular manifestations occur in 4 to 12% of IBD patients, with similar incidence in CD and UC [38].

Uveitis is the most common ocular manifestation, with a higher prevalence in CD (11.1%) than in UC (5.6%). In CD, the acute manifestation of the disease is concomitant with the intestinal disease flare, while in UC, this tight correlation does not exist [39]. Uveitis can be classified into four types: anterior, intermediate, posterior, and panuveitis based on the structures involved. Anterior uveitis affects the iris and the ciliary body and presents with conjunctival redness and photosensitivity. Intermediate and posterior uveitis are rarer and involve the vitreous and the retina, respectively, which can lead to permanent vision loss [38]. For this reason, suspected cases of uveitis require immediate referral to an ophthalmologist.

Episcleritis is an inflammation of the vascular layer between the sclera and conjunctiva that may involve the entire layer or a localized area [38], and it generally presents with hyperemia, pruritus, and burning [38]. The prevalence in CD is 3.95 [95% CI: 2.91–4.99] per 1000 patients and 4.73 [95% CI: 3.95–5.52] per 1000 patients in UC [40]. Typically, episcleritis occurs during IBD flares and can be diagnosed by the instillation of a topical vasoconstrictor [38].

Scleritis, an acute inflammation of the sclera, is a rare EIM in patients with IBD, although its incidence is increased compared to the general population (OR: 3.6; *p* < 0.001 in CD and OR: 2.2; *p* < 0.001 in UC) [41]. It can be classified as diffuse, nodular, and/or necrotizing, based on clinical manifestation, and anterior or posterior, based on location [42]. Unlike episcleritis, scleritis is not associated with intestinal disease flare-ups, and the vessels of the sclera do not whiten after the administration of vasoconstrictors, resulting in eye redness and intense pain, typically at night. Considering the risk of vision loss, an ophthalmologist should immediately be consulted, although evidence-based treatments are not available [1,43].

### 3.5. Hepatobiliary EIMs

PSC and autoimmune hepatitis (AIH) are immune-related liver disorders that can occur in almost 5% of IBD patients [44].

PSC is a condition characterized by the inflammation and fibrosis of the bile ducts that could culminate in end-stage liver disease [45]. The pooled prevalence in IBD is 5.01%, and it is more frequently associated with UC, 2.47%, than CD, 0.96% (OR 1.69, 95% CI 1.24–2.29) [46]. The male gender, pancolitis, non-smoking, and prior appendectomy are also risk factors for PSC [47]. Although PSC could be suspected based on elevated cholestatic liver enzymes, magnetic resonance cholangiopancreatography (MRCP) is the method of choice for the diagnosis [48]. Liver biopsy should be reserved for cases in which AIH overlap or small ducts PSC (negative MRCP) are suspected [45]. Importantly, patients with PSC and IBD should undergo yearly colonoscopy due to the 7-fold increased risk of developing colorectal cancer associated with PSC [49].

AIH occurs more frequently in patients with UC than in those with CD (OR 8 and 4, respectively) [50]. This condition should be suspected when liver enzyme levels are elevated after ruling out other causes, such as viral, alcoholic, and metabolic hepatitis. The liver biopsy is the definitive diagnostic test for AIH, presenting with features of ‘interface hepatitis’ on liver histology [51]. Additionally, MRCP should be performed due to the overlap syndrome with PSC (10%) [52].

### 3.6. Other EIMs

Patients with IBD are at an increased risk of developing venous thromboembolic events (VTE), which include deep vein thrombosis (DVT), splanchnic VTE, and pulmonary embolism [53], and the risk increases significantly during periods of disease flare-up [54]. There is no variation in the overall risk of thrombosis between genders or among patients with UC or CD [2]. Numerous studies on VTE have identified risk factors associated with IBD, including disease activity, disease phenotype, hospitalization, surgery, and corticosteroid usage, and factors unrelated to IBD, such as a history of thrombosis, obesity, pregnancy, and contraceptives [2,54]. In addition, the therapies currently employed in the treatment of IBD may be associated with an increased risk of VTE. Cases of VTE have been observed during JAK inhibitors (JAKi) trials and, although not specifically designed to assess VTE, an increased incidence of VTE events was observed in the oral surveillance trial [55]. On the other hand, two extensive meta-analyses involving patients exposed to JAKi across various immune-mediated inflammatory diseases (IMIDs) did not identify a heightened risk of VTE [56,57,58], and neither vedolizumab (VDZ) nor ustekinumab (UST) exhibited a heightened risk of VTE [59,60]. Anti-TNF agents may potentially reduce the risk of VTE in patients with IBD [4,58]. Nevertheless, it is recommended that all patients with IBD who are hospitalized for any reason, such as disease exacerbation or surgery, should be administered pharmacological thromboprophylaxis [2].

**Figure 2:**
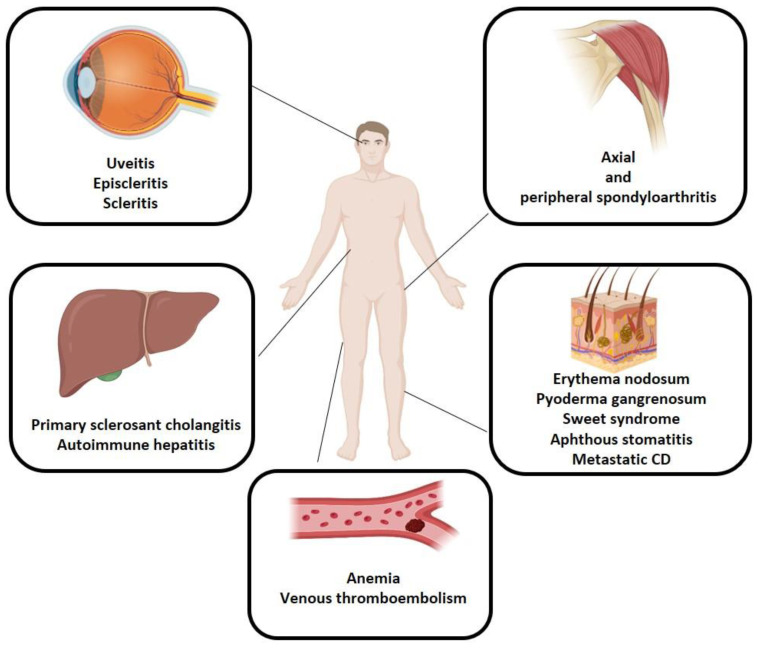
Main extraintestinal manifestations observed in patients with inflammatory bowel disease.

Anemia (defined as a hemoglobin level < 13 g/dL in men or <12 g/dL in non-pregnant women) is a prevalent EIM of IBD, with an approximate prevalence of 27% among individuals with CD and 21% among those with UC [61]. Iron deficiency and anemia of chronic disease are the predominant types observed in IBD [2]. The basic evaluation comprises red blood cell indices, such as red cell distribution width (RDW) and mean corpuscular volume (MCV), reticulocyte count, a complete blood count with differential, serum ferritin, transferrin saturation (TfS), and the concentration of C-reactive protein (CRP) [62]. A more comprehensive framing should include serum levels of vitamin B12, folic acid, haptoglobin, the proportion of hypochromic red cells, reticulocyte hemoglobin, lactate dehydrogenase, soluble transferrin receptor, creatinine, and urea [62]. For individuals with clinically active IBD, intravenous iron is the recommended initial treatment option due to its faster and more profound response, superior tolerance, and efficacy compared to oral iron supplementation [63]. Erythropoietin-stimulating agents may be contemplated for patients who exhibit an insufficient response to intravenous iron therapy [64], while intramuscular hydroxocobalamin represents the optimal intervention for the replacement of vitamin B12 in patients presenting with anemia resulting from micronutrient deficiencies [65]. Considering that anemia of chronic disease arises from reduced erythropoiesis, triggered by elevated levels of proinflammatory cytokines, the treatment of IBD may enhance bone marrow function [62,66] (Figure 2).

### 3.7. Treatments for EIMs: An Overview

Despite the high incidence of EIMs in patients with IBD, the data available on treatments are largely derived from retrospective, open-label studies and post-hoc analyses of RCTs.

Anti-TNFα are the most commonly utilized biologics in IBD and can also treat the majority of EIMs [2]. A shared effector phase is observed in immune-mediated inflammatory diseases, wherein bone marrow–derived macrophages, polymorphonuclear neutrophils, and fibroblasts are activated at the sites of inflammation, resulting in the production of TNF-α [17]. The only RCT that has been conducted thus far evaluates the use of infliximab (IFX) in PG. In the IFX group, there was a rapid and significant clinical response, with 46% of patients improving within two weeks compared to 6% in the placebo group. Overall, the response rate to IFX was 69%, with a remission rate of 21% when considering that at weeks 4 and 6, patients received open-label IFX [67]. In general, one of the most comprehensive studies on the utilization of anti-TNFα in EIMs was conducted by a Swiss cohort, which included 1249 patients, of whom 366 had at least one EIM. Of these patients, 213 (58.2%) were treated with an anti-TNFα. A clinical improvement of the EIM was observed in 71.8% of all anti-TNFα therapies. In 26.4% of cases, no response was observed, and in 1.8% of cases, a worsening of the EIM was observed. The highest response rates were observed in patients with psoriasis, aphthous stomatitis, uveitis, and peripheral arthritis, and there were no significant differences in response rates between different anti- TNFα agents [35]. With regard to the latter point, although there is a greater quantity of data available on the efficacy of IFX, adalimumab (ADA) can also be considered effective in the treatment of EIMs. In the CARE trial, 497 patients with EIMs who were treated with ADA demonstrated a 79% resolution of at least one EIM. The most efficacious outcomes were observed in the resolution of arthralgia (43%), arthritis (75%), and oral aphthous ulcers (59%) [68]. Fewer data are available for certolizumab pegol and golimumab: the approval of certolizumab pegol for RA, PA, and AS suggests that it may be efficacious in IBD-associated musculoskeletal manifestations [69,70,71], and it has also demonstrated efficacy in the treatment of uveitis [72]; the efficacy of golimumab in the treatment of musculoskeletal EIMs and acute uveitis is only known indirectly [73,74,75,76].

Concerning anti IL-12 and IL-23, a post hoc analysis of the UNITI trial, which included 504 patients with CD, demonstrated that there were no statistically significant differences between the UST and placebo groups at weeks 6 and 52 in terms of overall EIM resolution (36.9% vs. 39.1%, *p* = 0.564 and 76.4% vs. 80.0%, *p* = 0.542, respectively) [77]. In a recent systematic review, which included 152 IBD patients with EIMs, UST was found to be effective for arthralgia, PA, psoriasis, PG, while it exhibited poor efficacy in the axial SpA [78]. UST also demonstrated favorable outcomes in the treatment of psoriasis in a retrospective multicenter study, with a nearly 80% clinical resolution of the disease [79]. No data are currently available regarding the newly approved IL-23 inhibitors, Risankizumab, and mirikizumab.

The selective activity of VDZ on integrin α4β7, which is expressed only in the gut system, makes this drug ineffective for the treatment of pre-existing EIMs. However, there are some encouraging outcomes, indicating a lower occurrence of new EIMs under VDZ treatment [80]. Low likelihood of developing new episodes of arthralgia/arthritis in patients with CD was demonstrated in a post-hoc analysis of the GEMINI studies (VDZ vs. placebo: HR, 0.55; 95% CI 0.36–0.84). Conversely, in UC patients new/worsening arthritis/arthralgia had a similar incidence across treatment with VDZ and placebo (VDZ vs. PLA: HR, 0.99; 95% CI, 0.52–1.90) [81]. A real-life study on the use of UST and VDZ in EIMs confirmed the safe use of these drugs in patients with articular EIMs. Only a transient increased risk of developing new arthralgia was found after 6 months of VDZ treatment (VDZ 36/508 and UST 9/286; *p* = 0.021) [82].

In addition to anti-TNFα, JAKis have been demonstrated to be the most efficacious drugs for the treatment of EIMs due to their extensive spectrum of action. In a post-hoc analysis from OCTAVE trials, in both OCTAVE induction studies 1 and 2, comparable percentages of patients with active peripheral arthritis who received either tofacitinib or a placebo experienced either no change or some improvement. Specifically, 81.3% of the patients in the tofacitinib group and 85.7% of the patients in the placebo group exhibited no change or improvement, while 15.6% of the patients in the tofacitinib group and 14.3% of the patients in the placebo group demonstrated some improvement. However, by week 52 of OCTAVE Sustain, only the patients treated with tofacitinib exhibited improvements in active peripheral arthritis (16.7% and 33.3% of those receiving tofacitinib 5 mg and 10 mg twice daily, respectively) [83]. A review of the literature, which included 23 studies of tofacitinib in CD and UC, concluded that tofacitinib is an effective treatment for EIMs (dermatological, musculoskeletal, hepatological, and ophthalmological EIMs), particularly when used at higher dosages and in UC patients [84]. A recent abstract by Colombel et al. presents the findings of a pooled analysis from the U-ACHIEVE and U-ACCOMPLISH trials. The abstract demonstrates the efficacy of UPA in controlling EIMs in patients with UC: at induction (week 8), the delta in the resolution of any EIM, arthropathy, and anemia between UPA and placebo were 6.7%, 12.6%, and 5.6%, respectively. A greater difference was reached during maintenance, at week 52 (resolution of all EIM in 24.3% placebo, 41.7% UPA 15 mg, and 65.9% UPA 30 mg; *p* < 0.001, resolution of arthropathy in 22.2% placebo, 38.5% UPA 15 mg, and 66.7% UPA 30 mg; *p* < 0.01, and resolution of anemia in 36.4% placebo, 50.0% UPA 15 mg, and 70.8% UPA 30 mg; *p* < 0.019) [85] (Table 2).

#### 3.7.1. Treatment of Musculoskeletal EIMs

When managing axSpA in patients with IBD, there are currently no randomized controlled trials assessing the effectiveness of biologics, JAKis, or NSAIDs (non-steroidal anti-inflammatory drugs) specifically for this condition [86]. TNFα antagonists have been shown to reduce SpA associated with IBD [87,88,89,90]. Etanercept, a recombinant DNA-derived compound derived from the human immunoglobulin G1 and a TNFα antagonist, is a commonly used treatment for SpA, although it may induce paradoxical gastrointestinal inflammation and should therefore be avoided in the IBD population [91]. Considering the effectiveness of JAKis in treating AS, they may also be an option for managing axial SpA associated with IBD [92,93]. Conversely, VDZ, UST, and risankizumab are not recommended for managing axSpA in IBD [78,82,86,94]. A Sicilian cohort of 30 CD patients with SpA treated with UST demonstrated that 33.3% of patients achieved an articular response at week 8, and 43.3% at week 24. Notably, all resolution cases concerned peripheral SpA, suggesting that UST is ineffective in axial SpA [95].

Although the key trials of TNFα antagonists in IBD were not powered to assess the treatment of EIMs, the effectiveness of TNFα antagonists in treating peripheral arthritis is supported by the results of two open-label trials [68,88]. The CARE trial included 975 patients with CD who were treated with ADA. By week 20, the incidence of arthritis had decreased from 8.7% to 2.1% in comparison to the baseline [68]. Similarly, Generini et al. demonstrated a significant decrease in the incidence of arthritis (from 58% to 12.5%) in 24 patients with CD treated with IFX [88]. A systematic review of patients treated with UST for arthralgia or arthritis (nine studies included, 254) demonstrated its effectiveness in psoriatic arthropathy and arthralgia [78]. Concerning VDZ, a recently published multicenter retrospective cohort study showed an increased risk of new-onset arthralgia (adjusted OR: 2.28; 95% CI: 1.01–5.15; *p* = 0.047) [82].

#### 3.7.2. Treatment of Cutaneous EIMs

EN should be controlled by an effective IBD therapy (anti-TNF, UST) [35,68,96,97]. Symptomatic supportive care is usually sufficient for most patients [98]. In severe or refractory cases, systemic corticosteroids (prednisone 20 mg for 7–10 days) may be used [98].

Regarding PG, mild cases can be treated with local or topical therapies, such as intralesional corticosteroid injections, although systemic steroids are often necessary during the acute phase (prednisone 0.5–2 mg/kg/day, prednisolone 0.75 mg/kg/day) [99,100]. Treatment with biologic agents, particularly IFX and ADA, has been reported as effective in improving PG [67]. Recent data also suggests the effectiveness of UST and tofacitinib [101,102].

#### 3.7.3. Treatment of Ocular EIMs

After excluding the infectious causes of uveitis, topical steroids are the first-line of treatment for anterior uveitis, while systemic steroids are the preferred therapy for intermediate, posterior, and panuveitis [38]. Historically, medications such as AZA, cyclosporine, and cyclophosphamide have been used as second-line therapies for ocular inflammation that is unresponsive to steroid treatment [103,104]. However, the use of biologic therapy has significantly changed the way ocular EIMs are managed. ADA has shown to be effective compared to a placebo in three randomized control trials (HR: 0.50; 95% CI: 0.36–0.70; *p* < 0.001) [105,106,107]. Additionally, in a Swiss IBD cohort study it was found that 72% of individuals with uveitis exhibited a positive response to treatment with either IFX or ADA [35]. UST and tofacitinib have also been successfully used in treating uveitis [108,109].

Since episcleritis is a self-limiting condition, the treatment focuses on providing symptomatic relief and conservative measures. These measures include using topical lubricants such as artificial tears [110]. Additional topical NSAIDs or corticosteroids may be considered if symptoms persist or if achieving resolution of intestinal inflammation proves challenging [111].

COX-2 (cyclooxygenase-2) inhibitors and topical NSAIDs may be effective in mild forms of scleritis, while systemic corticosteroids are usually necessary, particularly in cases of posterior and necrotizing scleritis. Methotrexate (MTX), AZA, mycophenolate mofetil, calcineurin inhibitors, and IFX (5 mg/kg) are considered second-line therapy [112,113].

#### 3.7.4. Treatment of Hepatobiliary EIMs

In PSC, corticosteroids or biologics such as VDZ, ADA, and IFX have not been shown to benefit the progression of the hepatic disease [80,114,115]. Ursodeoxycholic acid at the dose of 15–20 mg/kg/day can improve liver biochemistry, but it does not improve symptoms, the risk of cholangiocarcinoma, or mortality [116]. Liver transplantation should be considered for patients with advanced cirrhosis, recurrent cholangitis (when the palliative treatment of bile duct stenosis with endoscopic retrograde cholangiopancreatography has failed), and suspected early-stage biliary neoplasia [45]. Almost 50% of patients with PSC will require a liver transplant 15–20 years after diagnosis [117], and the overall survival rate after liver transplantation is excellent, with a 91% survival rate at 1–5 years post-transplant [118].

Systemic steroids are the first-line therapy for the AIH acute phase, while AZA is the preferred maintenance therapy. In cases of refractory disease, mycophenolate, mercaptopurine, or IFX may be considered as second-line treatment options [52,119,120].

#### 3.7.5. Paradoxical Effects of Advanced Therapies

One crucial aspect to be taken into account in the treatment of IBD is the paradoxical effects of drugs that can be defined as the onset or worsening of a pathological condition, which typically responds to this class of drug, occurring while treating a patient for a different condition [121]. The precise mechanism by which this phenomenon occurs is not fully understood. However, it is thought that genetic predisposition, the development of antibodies against biological agents, and off-target effects may all contribute to the development of this effect [122,123]. Most of the data available on the paradoxical effects of drugs in IBD involve anti-TNF-α, with an estimated incidence of more than 10% [123]. The most common paradoxical effect observed in this class of drug is the appearance of skin manifestations, which may be psoriasis-like (1.6% to 2.7%) [124]. Skin lesions improve significantly after the discontinuation of TNF antagonists in most patients with basic hygiene practices and the use of topical treatments such as corticosteroids [123]. Other paradoxical manifestations include uveitis, vasculitis, lupus and exacerbation of IBD, CD-like [123]. Importantly, in a systematic review of 17 studies of TNFα-induced paradoxical psoriasis, switching to UST resulted in complete or partial resolution of psoriasis in 83.1% of patients (n = 89). In comparison, switching to VDZ resulted in complete resolution in 100% of patients (n = 6) [125]. Regarding other drug classes, the incidence of paradoxical effects associated with other advanced therapies is minimal: UST might cause new-onset or worsening of preexisting psoriatic lesions or other autoimmune conditions such as lupus-like syndrome [126]; JAKis have been associated with a slight increase in the risk of viral infections due to immunosuppression. For instance, the incidence of varicella-zoster virus infection has been reported to be higher with tofacitinib 10 mg (RR = 6.90; 95% CI 1.56–30.63) and upadacitinib 45 mg (RR = 7.89; 95% CI 1.04–59.59). In addition, the incidence of VTE, including DVT and pulmonary embolism, was 0.68 (95% CI 0.36–1.29), 0.44 (95% CI 0.28–0.70), and 0.59 (95% CI 0.31–1.15), respectively [127]. Additionally, there was a potential exacerbation of other autoimmune diseases and hyperlipidemia [56,128]. VDZ paradoxical effects are less common and can present with musculoskeletal symptoms in up to 30% of cases, although only half of patients had actually arthritis. Exacerbation of IBD, new-onset of psoriasis or eczema, or uveitis occur in <1% of cases [19,129]. These paradoxical reactions often improve when the medication is discontinued or switched to another agent [121].

## 4. Discussion

According to the most recent definition, EIMs are inflammatory conditions occurring beyond the gastrointestinal tract, where their development is either influenced by the spread of immune responses from the intestine, or it is a separate inflammatory process sustained by IBD, or they share similar genetic or environmental factors with IBD [8]. Other extraintestinal conditions may be considered complications of the disease, or the therapies given, or associated conditions with an uncertain mechanism [8].

In general, patients with IBD have a higher risk of developing other immune-mediated disease, and these are more frequent in patients with CD than UC (OR 1.48, 95% CI 1.08–1.87) [130]. In addition, EIMs occur before the diagnosis of IBD in almost 15% of cases, and in these cases, IBD has a more aggressive pattern [130] Large amount of data regarding epidemiology are obtained from the ENEIDA registry, which includes 31,077 patients with IBD. Almost 19% of these patients had at least one EIM. The registry confirms that musculoskeletal manifestations are the most frequent EIM, with a prevalence of 13% (95% CI 12.9–13.7). Cutaneous manifestations follow, with a prevalence of 5% (95% CI 4.7–5.2), while ocular manifestations and hepatobiliary manifestations have a prevalence of 2.1% (95% CI 1.9–2.2) and 0.7% (95% CI 0.6–0.8), respectively [131].

To date, the most commonly used therapies for patients presenting with EIMs are anti-TNF-α [132]. The efficacy of this class of drugs may be attributed to the fact that a common pathway of inflammation in a variety of immune-mediated diseases is the activation of TNF-α [17]. Similarly, JAKis, which exhibit a broad spectrum of action, are also efficacious pharmaceutical agents in the treatment of various types of EIMs [84]. However, they should be employed with greater caution in patients aged 65 years and older, as well as in those with a history of cardiovascular disease, in accordance with the warnings issued by the EMA and FDA [55]. In addition, the TENOR study may be able to provide insight into the efficacy of UST in the treatment of EIMs [133]. It is also essential to be aware that certain medications utilized to treat IBDs may precipitate symptoms that are analogous to those observed in EIMs [4]. Consequently, distinguishing between the etiology of symptoms is a significant challenge for both the gastroenterologist and the multidisciplinary team.

As we look to the future, it seems clear that in order to improve treatment and thus the quality of life of these patients, more and more efforts should be made to better define the etiopathogenesis of these diseases and classify them, as had already been proposed, not by organ but by the mechanism underlying the disease [17]. Furthermore, it would be beneficial to conduct prospective, randomized studies and head-to-head trials with the resolution of EIMs as an end-point, as currently, only retrospective studies and post-hoc analyses have been conducted.

Finally, a multidisciplinary approach involving collaboration with other specialists, such as rheumatologists, dermatologists, and hepatologists, is crucial to ensure high standard of care for patients with IBD, taking into account the complexity of all their comorbidities.

## 5. Conclusions

IBD are complex diseases that share pathogenetic mechanisms and environmental risk factors with other immune-mediated diseases. Therefore, they should not be interpreted as diseases limited to the gastrointestinal tract, but as systemic diseases requiring a multidisciplinary approach.

## Figures and Tables

**Table 1:** Genetic risk factors between inflammatory bowel disease and extraintestinal manifestations.

Genetic Risk Factors between Inflammatory Bowel Disease and Extraintestinal Manifestations	
Ankylosing spondylitis [15]	IL23R
Sacroiliitis [13]	NOD2/CARD15
Erythema nodosum [16]	PTGER4, ITGAL, SOCS5, CD207, ITGB3, rs6828740
Pyoderma gangrenosum [16]	IL8RA, PRDM1, USP15, TIMP3
Uveitis [14]	NOD2/CARD15
Primary sclerosing cholangitis [1]	SOCS1, JAK2, STAT3, TYK2, UBASSH3A, BCL2L11, FOXO1, IRF1

**Table 2:** Treatments of extraintestinal manifestation in inflammatory bowel disease.

System Involved	Disease	Synchronous with IBD	Treatment That Should Be Considered	Treatment That May Be Considered	Treatment That Cannot Be Recommended
**Musculoskeletal EIM** **13% (12.9–13.7)**	Ankylosing spondylitis	no	Anti TNF-α	JAKi	vedolizumab, ustekinumab
	Sacroiliitis	no	Anti TNF-α	JAKi	vedolizumab, ustekinumab
	Peripheral arthritis type 1	yes	Anti TNF-α, JAKi, ustekinumab	vedolizumab	-
	Peripheral arthritis type 2	no	Anti TNF-α, JAKi, ustekinumab	vedolizumab	-
**Skin EIM** **5% (4.7–5.2)**	Erythema nodosum	yes	-	Anti TNF-α	vedolizumab
	Pyoderma gangrenosum	yes or no	systemic steroids	Anti TNF-α, JAKi, ustekinumab	vedolizumab
**Ocular EIM** **2.1% (1.9–2.2)**	Uveitis	yes or no	topical steroids, anti TNF-α (100%)	JAKi, ustekinumab	vedolizumab
	Episcleritis	yes	topical lubricants	NSAIDs, corticosteroids	-
	Scleritis	yes or no	COX-2 inhibitors, topical NSAIDs, systemic corticosteroids	MTX, AZA, mycophenolate mofetil, calcineurin inhibitors, IFX	-
**Hepatobiliary EIM** **0.75% (0.6–0.8)**	Primary sclerosing cholangitis	no	-	Ursodeoxycholic acid	-
	Autoimmune hepatitis	yes or no	Systemic steroids, AZA	infliximab	-

IBD: inflammatory bowel disease; EIM: extraintestinal manifestation; TNF-α: tumor necrosis factor- α; JAKi: Janus kinase inhibitor; NSAIDs: non-steroidal anti-inflammatory drugs; COX-2 inhibitors: Cyclooxygenase-2 inhibitors; MTX: methotrexate; AZA: azathioprine.

## Data Availability

No new data were generated or analyzed in support of this research.
